# Protocol for differentiating murine 3T3-L1 and SVF-derived preadipocytes and isolating crude mitochondrial fractions

**DOI:** 10.1016/j.xpro.2025.104045

**Published:** 2025-08-23

**Authors:** Churaibhon Wisessaowapak, Jeongmin Lee, Hyeonhui Kim, Seunghwan Son, Xue Feng, Lina Chang, Annie Hoang, Hetty Chen, Sarah Bedsted, Alan R. Saltiel

**Affiliations:** 1 Division of Endocrinology and Metabolism, Department of Medicine, University of California, San Diego, San Diego, CA, USA; 2 Department of Pharmacology, University of California, San Diego, San Diego, CA, USA

**Keywords:** Cell culture, Metabolism, Molecular Biology

## Abstract

Here, we present a protocol for differentiating 3T3-L1 preadipocytes and stromal vascular fraction (SVF)-derived preadipocytes from mice into mature adipocytes, followed by the isolation of crude mitochondrial fractions. This cost-effective and reproducible protocol is optimized for small-plate formats, compatible with standard reagents, and suitable for metabolic studies such as insulin resistance and mitochondrial function.

## Highlights

• Culture of mouse 3T3-L1 fibroblasts
• Isolation of primary preadipocytes from mouse iWAT
• Differentiation of mouse 3T3-L1 and SVF-derived preadipocytes into adipocytes
• Isolation of crude mitochondrial and cytosolic fractions from mouse adipocytes

## Abstract (editor highlights)

Publisher’s note: Undertaking any experimental protocol requires adherence to local institutional guidelines for laboratory safety and ethics.

## Graphical abstract

**Figure:**
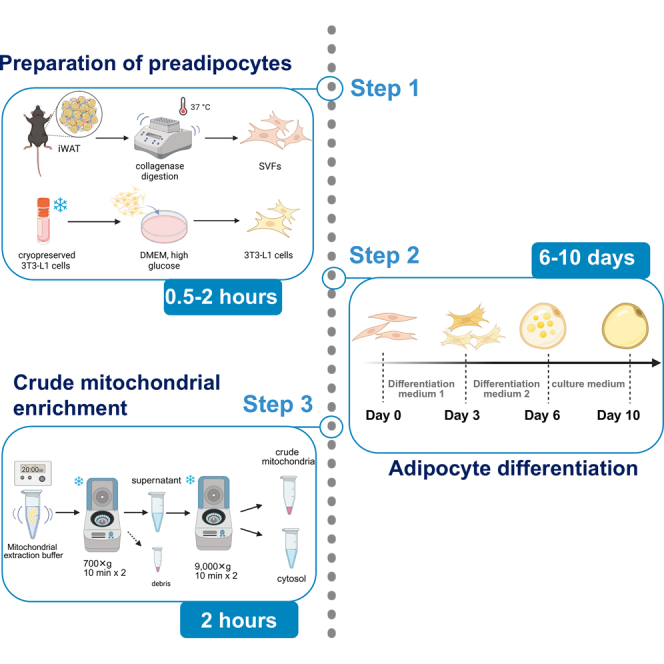

## Before you begin

Adipocyte differentiation models, such as 3T3-L1 preadipocytes and SVF-derived primary cells, are indispensable for investigating metabolic signaling, lipid homeostasis, and mitochondrial dynamics.^1^ These systems offer robustness, reproducibility, and scalability, enabling researchers to dissect molecular mechanisms underlying obesity, insulin resistance, and related metabolic disorders.

Despite their utility, isolating mitochondria from adipocytes presents unique technical challenges due to the cells’ lipid-rich cytoplasm, fragile organelle membranes, and typically low mitochondrial yield compared to brown or beige adipocytes.^2^ While commercial mitochondrial isolation kits are available, they are often costly and may not be optimized for adipocyte-specific applications.

This protocol details an efficient and cost-effective method for the in vitro differentiation of both the established 3T3-L1 preadipocyte cell line and primary preadipocytes derived from SVF. It then outlines a procedure for crude mitochondrial fractionation specifically optimized for these differentiated adipocytes. The resulting mitochondrial isolates are suitable for a range of downstream applications, including Western blotting and qPCR. This method prioritizes affordability, speed, and efficacy, offering a valuable alternative to more expensive or time-consuming approaches.

To ensure reproducibility and success, several preparatory steps are essential. First, obtain 3T3-L1 preadipocytes at a low passage number (ideally <15) and maintain them using best practices to preserve differentiation potential. Use high-quality, endotoxin-tested serum and prepare fresh differentiation media components with proper storage to ensure batch-to-batch consistency. A sterile workspace and proper aseptic technique are critical for both cell culture and subcellular fractionation. Finally, this protocol employs basic differential centrifugation for crude mitochondrial isolation, assessing whether the resulting purity meets the requirements for your intended downstream applications.

### Innovation

This protocol introduces key refinements that improve the efficiency and accessibility of adipocyte-based mitochondrial studies. It enables adipocyte differentiation in small plate formats, reducing reagent use and increasing throughput. For SVF isolation, the protocol minimizes collagenase volume by using 1 mg/mL in just 1–1.5 mL per mouse, cuts digestion time in half from 30 minutes to 15 minutes, significantly lowering costs and experimental time without compromising yield. Crucially, it allows crude mitochondrial isolation directly from monolayer cultures using only standard centrifugation and a simple lysis buffer, avoiding the need for Dounce homogenizers, ultracentrifuges, or commercial kits. These modifications make the workflow particularly well-suited for high-throughput studies and resource-limited settings. By integrating these practical optimizations, this protocol expands access to adipocyte mitochondrial studies in metabolic research.

### Institutional permission

All animal procedures were approved by the Institutional Animal Care and Use Committee (IACUC) at the University of California, San Diego, under protocol number S15176. Researchers intending to use this protocol must obtain prior approval from their respective institutional oversight committees.

### Thawing and initial culture of cryopreserved 3T3-L1 cells

**Timing: 30 min**

This procedure outlines the rapid thawing of cryopreserved 3T3-L1 preadipocytes and the initiation of their culture. The growth medium is supplemented with newborn calf serum (NBCS) to reduce the rate of proliferation^3^ and effectively maintain the cells in an undifferentiated, fibroblastic state.

1. Preparation for growth medium and culture vessel.
  a. Prepare NBCS medium.
  b. Add an appropriate volume (e.g., 10 mL for a 10 cm diameter dish or 22 mL for a 15 cm diameter dish) of the NBCS medium to a sterile dish. Allow the medium to warm in a cell culture incubator at 37°C, 10% CO_2_.
2. Rapid thawing of cryovial.
  a. Retrieve the cryovial containing the frozen 3T3-L1 cells from liquid nitrogen or a −80°C freezer.
  b. Immediately immerse the cryovial in a 37°C water bath.
  c. Gently swirl the cryovial continuously until no ice crystals remain. Thawing should be rapid and completed within 1–2 minutes to minimize ice crystal-induced cell damage.
3. Centrifugation to remove cryopreservation medium.
  a. Prepare a 15 mL tube. Add 5 mL prewarmed NBCS medium to the tube.
  b. Remove the cryovial from the water bath and wipe it down with 70% ethanol to ensure sterility.
  c. Pipet the cell suspension from the cryovial and gently transfer it into a 15 mL tube.
  d. Centrifuge at 500 × g, 25°C for 5 minutes.
  e. Discard the medium and gently resuspend the pellet with 1 mL NBCS medium.
4. Transfer cells to the culture dish.
  a. Pipet the cell suspension to the plate from step 3e.
  b. Gently swirl the culture dish to evenly distribute the cell suspension in the medium.
  c. Place the culture dish in a humidified cell culture incubator maintained at 37°C and 10% CO_2_.
  d. Incubate the cells for 24 hours without disturbing them to allow them to attach to the culture surface.
5. Media Change and Subsequent Culture.
  a. After 24 hours of incubation, carefully aspirate the initial growth medium, which may contain residual DMSO and dead cells from the cryopreservation process.
  b. Gently add fresh, pre-warmed NBCS medium to the culture dish (same volume as in Step 1b).
  c. Return the dish to the incubator and continue culturing the cells, changing the growth medium every 2–3 days as needed, until they reach the desired confluency for passaging or differentiation experiments.

### Expansion and maintenance of 3T3-L1 cells

**Timing: 30 min**

This step is optimized for growing 3T3-L1 cells in a 15 cm dish.

6. Passaging 3T3-L1 Cells.
  a. Monitor cells under the microscope. When they reach 60%–70% confluency, proceed to passage.
  b. Aspirate the old medium and wash once with 5 mL warm PBS. Swirl the dish gently to ensure complete coverage and then aspirate the PBS.
  c. Add 3 mL of 0.25% trypsin-EDTA solution to the dish, ensuring the entire cell monolayer is coated.
  d. Incubate at 37°C, 10% CO_2_ for 5–10 minutes.
  e. Observe under the microscope to confirm detachment. Tap the side of the dish gently to aid detachment if necessary.
7. Neutralization and harvesting.
  a. Add 10 mL of complete medium to neutralize the trypsin activity.
  b. Gently pipette the cell suspension up and down 8–10 times using a 10 mL serological pipette to break up cell clumps and obtain a single-cell suspension.
  c. Transfer the entire cell suspension to a 15 mL or 50 mL sterile conical tube.
8. Centrifugation and resuspension.
  a. Centrifuge at 500 × g for 5 minutes at 25°C.
  b. Carefully aspirate and discard the supernatant without disturbing the cell pellet.
  c. Resuspend the cell pellet in 30 mL of fresh complete growth medium by gently pipetting up and down 8–10 times using a 10 mL serological pipette to ensure a homogeneous suspension.
9. Seeding cells for expansion.
  a. Add 22 mL of fresh medium to a new 15 cm dish.
  b. Transfer 1 mL of the resuspended cells from step 8c into the new plate.
  c. Swirl gently and incubate at 37°C, 10% CO_2_. Healthy 3T3-L1 preadipocytes typically reach 60%–70% confluency within 3–4 days.

**CRITICAL:** Avoid allowing cells to become over-confluent, as this can induce growth arrest in fibroblasts.^4^ Passage the cells regularly to maintain them in the preadipocyte state.

10. Seeding cells for experiments.
  a. Use the cell suspension prepared in Step 8c to seed cells at the desired density for differentiation experiments (see Table 1).

**Table 1:** Recommended seeding volumes for different plate and dish formats

Plate type	Cell suspension from step 8c (mL)	NBCS medium (mL)	Total volume (mL)	Volume per well (mL)
6-well	10	9	19	3
12-well	10	9	19	1.5
24-well	10	15	25	1
6 cm dish	3	2	5	5
10 cm dish	6	4	10	10

***Note:*** The seeding density will depend on the specific experimental design and the desired confluency at the start of differentiation.

***Note:*** Smaller well formats are often preferred for differentiation assays due to easier handling of reagents and potentially more uniform differentiation. For a 12-well plate, the addition of 150 μL of lysis buffer to each well after differentiation typically yields a protein concentration of approximately 2–3 μg/μL. For RNA extraction from a 12-well plate, eluting the RNA in 25 μL of RNase-free water often results in a concentration of around 500 ng/μL. For a 24-well plate, using 100 μL of lysis buffer typically yields a protein concentration of 1–1.5 μg/μL, and eluting RNA in 25 μL of RNase-free water can yield concentrations of 100–300 ng/μL. These yields can vary depending on cell density and lysis/extraction efficiency.

### Isolation of SVF for adipocyte differentiation

**Timing: 2 h for SVF isolation, 7 days for expansion**

This protocol describes the enzymatic digestion and isolation of SVF from iWAT of both male and female 8 to 10-week-old C57BL/6 mice, followed by seeding and in vitro adipogenic differentiation into primary preadipocytes (PPDIVs).

11. Dissection of iWAT (Figure 1).
  a. Euthanize the mouse according to the approved IACUC protocols.
  b. Thoroughly spray the fur of the mouse with 70% ethanol to minimize contamination.
  c. Make a lower abdominal incision to expose the iWAT pads bilaterally.
  d. Carefully dissect both iWAT pads using sterile forceps and scissors, ensuring the removal of any adjacent lymph nodes.
  e. Transfer the dissected tissue to a 10 cm sterile culture dish containing 5 mL of pre-warmed PBS with 100 U/mL Pen-Strep. Wash the tissue gently to remove blood, debris, and fur until the tissue appears clean.

**Figure 1:**
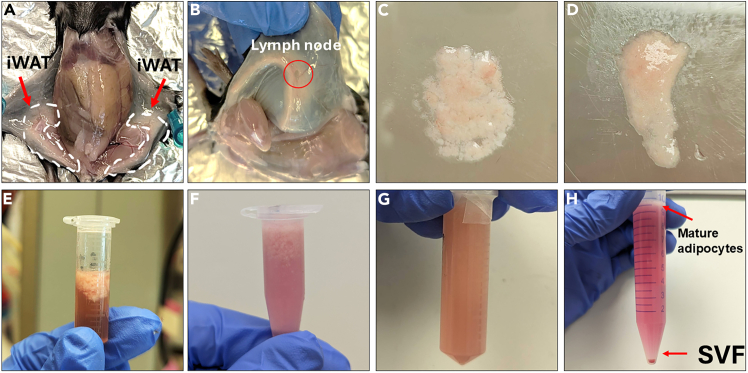
Isolation of SVF from iWAT of a C57BL/6 mouse via enzymatic digestion This figure illustrates the key steps involved in the isolation of SVF from the iWAT of a 10-week-old male C57BL/6 mouse using collagenase digestion. (A) Representative image of a mouse cadaver showing the location of iWAT pads (red arrows). (B) Close-up image of the dissected iWAT, highlighting a lymph node (red circle) that needs to be removed prior to tissue processing. (C) Incomplete minced tissue. (D) Well-minced tissue with no visible chunks. (E) iWAT tissue suspension before collagenase digestion. (F) Under digested tissue with some visible tissue chunks. (G) Tissue suspension after a complete digestion. No visible chunks appear. (H) Centrifuged cell suspension showing the separation of mature adipocytes (top, red arrow) and the SVF cell pellet (bottom, red arrow).
12. Enzymatic digestion.
  a. Transfer the washed iWAT tissue to a new 10 cm sterile culture dish. Mince the tissue finely using a sterile razor blade or scalpel until a homogenous slurry is obtained.
    **CRITICAL:** Thorough mincing of the adipose tissue is crucial for efficient collagenase digestion and optimal SVF yield. Inadequately minced tissue can result in lower cell recovery.
  b. Transfer the minced tissue slurry into a 1.5 mL microcentrifuge tube containing 1 mL of pre-warmed 1 mg/mL collagenase type I dissolved in DMEM/F12 without FBS.
    ***Note:*** For 1 mouse, using 1 mL of 1 mg/mL collagenase is enough to digest tissues.
  c. Incubate the tissue-collagenase mixture at 37°C in the Eppendorf ThermoMixer F2.0 at 1,000 RPM for 10–15 minutes. The tissue should be completely digested, with no visible chunks of adipose tissue remaining.
    ***Note:*** The optimal incubation time and shaking speed may vary depending on the type of shaker used. We have also successfully isolated SVF using a reciprocal water bath shaker at 37°C for 30 min, using 5 mL of 1 mg/mL collagenase type I dissolved in DMEM/F12, inverting the tube gently every 5 min.
13. Filtration and SVF Isolation.
  **CRITICAL:** Perform all steps in a biosafety level 2 hood to maintain sterility and prevent contamination.
  a. Add 5 mL of DMEM/F12 supplemented with 10% FBS (SVF medium) to a 15 mL conical tube.
  b. Pipet the digested tissue mixture from Step 12c to the tube to neutralize the collagenase activity. Mix gently by inverting the tube several times.
  c. Filter the entire digested tissue suspension through a 100 μm nylon mesh cell strainer into a new sterile 50 mL conical tube. Rinse the original tube with an additional 1 mL of SVF medium and pass this rinse through the same strainer to maximize cell recovery.
  d. Centrifuge the filtered cell suspension at 700 × g for 5 minutes at 25°C. Following centrifugation, the mixture will typically separate into three layers, a top layer of mature adipocytes (lipid-rich), the supernatant (containing digestion enzymes and medium), and a pellet at the bottom containing the SVF and other cell types.
  e. Carefully discard the top lipid layer and the supernatant using a low vacuum aspiration system or by gently pipetting. Avoid disturbing the cell pellet at the bottom.
  f. Resuspend the cell pellet in 10 mL of complete SVF medium and gently pipette up and down several times to obtain a homogenous suspension.
  g. Centrifuge the cell suspension at 700 × g for 5 minutes at 25°C. Carefully aspirate and discard the supernatant.
14. Seeding SVF for Expansion.
  a. Resuspend the SVF cell pellet in 1 mL of complete SVF growth medium using a P1000 micropipette. Gently pipette up and down approximately 5–10 times to ensure a single-cell suspension without any visible cell clumps.
  b. Seed the resuspended SVF cells into a 10 cm sterile culture dish containing 10 mL of SVF growth medium. Gently swirl the dish to distribute the cells evenly.
  c. Incubate the cells at least 12 hours, at 37°C with 10% CO_2_, to allow them to attach to the culture surface.
  d. The next day, carefully aspirate the medium to remove any non-adherent cells and debris. Wash the cells several times with warm PBS to further remove any remaining contaminants.
  e. Add fresh SVF medium to the dish and continue culturing the cells in an incubator at 37°C with 10% CO_2_. Allow the cells to proliferate until they reach the desired confluency (typically 80%–90%) for passaging or differentiation experiments. This usually takes 5–7 days after the initial seeding. Change the medium every 2 days.
15. Seeding Cells for Experiments.
  a. After the SVFs reach confluency, aspirate the culture medium and wash the cells once with 5 mL of warm PBS.
  b. Add 1 mL of 0.25% trypsin-EDTA solution to the dish to cover the cell monolayer.
  c. Incubate the dish at 37°C for 5 minutes, or until the cells have detached from the culture surface.
  d. Add 5 mL of complete SVF growth medium to the dish to neutralize the trypsin activity.
  e. Gently pipette the cell suspension up and down 5–10 times using a 10 mL serological pipette to obtain a single-cell suspension.
  f. Take a 10 μL aliquot of the cell suspension, mix with trypan blue and count the cells using a hemocytometer to determine the cell density.
  g. Calculate the required volume of cell suspension to seed the desired number of cells per well or dish for your experiment and resuspend the cells in complete SVF growth medium at a concentration of 1 × 10^6^ cells/mL (See Table 2).

**Table 2:** Seeding volumes and approximate cell numbers for various plate and dish formats

Plate type	Cell suspension (mL) for 1 × 10^6^ cells/mL	Approximate cell number
6-well	2	1 × 10^6^
12-well	1	5 × 10^5^
24-well	0.5	2.5 × 10^5^
6 cm dish	5	5 × 10^6^
10 cm dish	10	10 × 10^6^

***Note****:* SVF, like preadipocytes, may exhibit better differentiation potential when seeded at appropriate densities in smaller well formats. For a 12-well plate, lysis with 150 μL of lysis buffer after differentiation typically yields a protein concentration of approximately 1–2 μg/μL. For RNA extraction from a 12-well plate, elution with 25 μL of RNase-free water can yield RNA concentrations of around 100-300 ng/μL. For a 24-well plate, lysis with 100 μL of lysis buffer typically yields a protein concentration of 1–1.5 μg/μL, and RNA elution in 25 μL of RNase-free water can yield concentrations of 80–150 ng/μL. These yields can vary based on cell density and the efficiency of lysis and extraction procedures.

### Differentiation of 3T3-L1 preadipocytes and SVFs into adipocytes

**Timing: 9 days (2 days post-confluency + 7 days differentiation)**

This section describes a two-step induction protocol for differentiating confluent 3T3-L1 fibroblasts and SVFs into mature adipocytes, characterized by lipid droplet accumulation. The standard adipogenic cocktail initiates differentiation through two primary mechanisms, 3-isobutyl-1-methylxanthine (IBMX) and dexamethasone (Dexa) induce the expression of the transcription factors C/EBPδ and C/EBPβ, while insulin promotes glucose uptake for triacylglycerol synthesis.^5^ For primary cell differentiation, the SVF is additionally treated with troglitazone (TZD), a potent PPARγ activator, to further drive the adipogenic program.^6^

16. Induction of Differentiation (Day 0–3).
  a. Once 3T3-L1 cells or SVFs reach 100% confluency, allow them to continue culturing for an additional 2 days post-confluency (Day −2 to Day 0). This growth arrest is crucial for initiating uniform differentiation.
  b. On Day 0, prepare the differentiation medium I (DM I) using the FBS medium for 3T3L-1 cells or PPDIVs medium I (PM I) using the SVF medium for PPDIVs. The volume of media and supplements are in this table. The final concentration of each supplement is as follows: 1 μg/ml insulin, 250 nM dexamethasone, 500 μM IBMX, and 1 μM TZD (see Table 3).

**Table 3:** Volumes of differentiation reagents and medium for adipogenesis

Plate type	Volume per well (mL)	Total volume (mL)	1 mg/mL insulin (μL)	2.5 mM dexa (μL)	250 mM IBMX (μL)	10 mM TZD (μL)^a^
6-well	2.5	24	24	2.4	48	2.4
12-well	1.5	18	18	1.8	36	1.8
24-well	1	24	24	2.4	48	2.4
6 cm dish	6	6	6	0.6	12	0.6
10 cm dish	12	12	12	1.2	24	1.2

a Add TZD only to PM I for PPDIVs. Differentiation media should be freshly prepared on Day 0 but can be stored at 4°C for up to 1 week if necessary. However, using freshly prepared medium is recommended for optimal differentiation efficiency.
  c. Carefully aspirate the growth medium from the confluent cells.
  d. Gently add the freshly prepared DM I or PM I to each well or dish according to the volumes specified in the table below, dispensing the medium along the side of the well to avoid disturbing the cell monolayer.
  e. Incubate the cells at 37°C with 10% CO_2_ for 3 days.
17. Switch to Maintenance Medium (Day 3–6).
  a. On Day 3, prepare the differentiation medium II (DM II) using the FBS medium for 3T3-L1 cells or PPDIVs medium II (PM II) using the SVF medium for PPDIVs. The final concentration of insulin in DM II and PM II is 1 μg/mL (See Table 4).

**Table 4:** Volumes of insulin and medium for adipogenesis

Plate type	Volume per well (mL)	Total volume (mL)	1 mg/ml insulin (μL)
6-well	2.5	24	24
12-well	1.5	18	18
24-well	1	24	24
6 cm dish	6	6	6
10 cm dish	12	12	12
  b. Gently aspirate the medium from the cells. The medium may appear more viscous and yellowish.
    **CRITICAL:** Differentiating adipocytes are fragile and can detach easily. Use extreme caution during aspiration, employing a P1000 micropipette with gentle suction or a low vacuum aspiration system with a P10 tip to avoid disrupting the cell layer.
  c. Gently add the medium to each well or dish according to the volumes in table on step 16b, dispensing the medium along the wall of the well to minimize disturbance to the differentiating cells.
  d. Incubate the cells at 37°C with 10% CO_2_ for an additional 3 days.
18. Maturation and Maintenance (Day 6 onwards).
  a. On Day 6, gently aspirate the medium out.
  b. Replace the medium with the standard growth medium for long-term maturation (FBS medium for 3T3-L1 cells and SVF medium for PPDIVs). At this stage, small lipid droplets should become visible within the adipocytes under a microscope, and these will accumulate over time (Figures 2 and 3).

**Figure 2:**
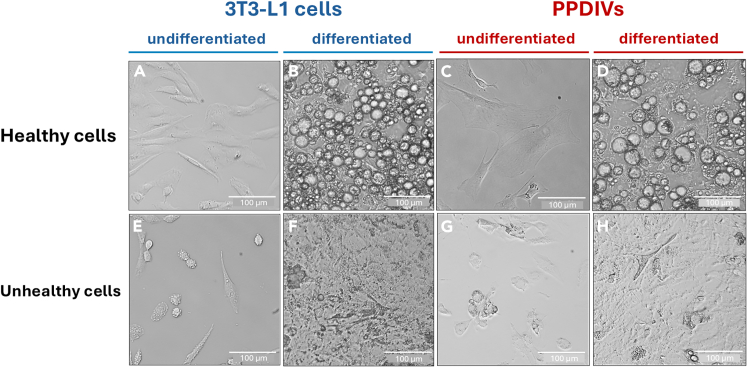
Bright-field images of undifferentiated and differentiated adipocytes from 3T3-L1 cells and PPDIVs (A and C) Representative images of healthy undifferentiated 3T3-L1 cells and primary preadipocytes (PPDIVs), displaying fibroblast-like morphology with elongated, spindle-shaped structures. (B and D) Differentiated 3T3-L1 and PPDIVs exhibiting typical adipocyte morphology characterized by rounded cells with visible intracellular lipid droplets. (E and G) Morphologically unhealthy undifferentiated 3T3-L1 cells and PPDIVs showing irregular shapes, cytoplasmic condensation, and loss of adherence. (F and H) Representative images of unhealthy differentiated 3T3-L1 cells and PPDIVs showing poor adipogenic morphology with no visible lipid accumulation. Scale bars, 100 μm. Images captured at 20× magnification.
  c. Continue culturing the cells in the standard growth medium, changing the medium every 2 days as needed, for up to Day 10 or longer depending on the experimental requirements.

***Note:*** For optimal results in metabolic studies, differentiated adipocytes are typically used for experiments between day 7 and day 8 of differentiation. Adipocytes at day 6 can be used for procedures like siRNA transfection, as they have begun the differentiation process but may be more amenable to transfection.

## Key resources table

**Table:** 

REAGENT or RESOURCE	SOURCE	IDENTIFIER
**Antibodies**		

VDAC (1:2,000 dilution)	Cell Signaling Technology	Cat# 4661, RRID:AB_10557420
COX IV (1:1,000 dilution)	Cell Signaling Technology	Cat# 4850, RRID:AB_2085424
TOM 20 (1:1,000 dilution)	Cell Signaling Technology	Cat# 42406, RRID:AB_2687663
β tubulin (1:3,000 dilution)	Cell Signaling Technology	Cat# 2146, RRID:AB_2210545
β actin (1:3,000 dilution)	Cell Signaling Technology	Cat# 4967, RRID:AB_330288
Calnexin (1:1,000 dilution)	Thermo Fisher Scientific	Cat# PA5-34754, RRID:AB_2552106
Goat anti-rabbit IgG secondary antibody (1:5,000 dilution)	Invitrogen	Cat#G-21234
Donkey anti-goat IgG (H+L) secondary antibody (1:5,000 dilution)	Invitrogen	Cat#A15999

**Chemicals, peptides, and recombinant proteins**		

Collagenase type 1	Sigma-Aldrich	SCR103
Fetal bovine serum	Omega Scientific	FB-01
Newborn calf serum	Gibco	16010–159
Phosphate-buffered saline (PBS) + Ca^2+^, +Mg^2+^	Corning	21-030-CM
DMEM	Gibco	11995–065
DMEM/F-12	Gibco	11320033
10,000 U/mL Pen-Strep	Gibco	15070–063
0.25% Trypsin-EDTA	Gibco	25200–056
Trypan blue stain	Invitrogen	T10282
Insulin	Sigma-Aldrich	I-5523
Dexamethasone	Sigma-Aldrich	D-1756
3-isobutyl-1-methylxanthine	Sigma-Aldrich	I-1578
Troglitazone	Sigma-Aldrich	T2573
HEPES pH 7.8	Sigma-Aldrich	H3375
NaCl	Sigma-Aldrich	71376
MgCl_2_	Sigma-Aldrich	M8266
Digitonin	Sigma-Aldrich	D141
EDTA	Invitrogen	AM9260G
EGTA	EG Scientific	E−2491
RIPA	Cell Signaling Technology	9806
Protease Inhibitor Cocktail	Millipore	539132
Phosphatase Inhibitor Cocktail	Sigma-Aldrich	P0044
4x Laemmli Sample Buffer	Bio-Rad	1610747
Novex Tris-Glycine Mini Protein Gels, 4%–20%	Invitrogen	XP04205BOX
Immun-Blot PVDF membrane	Bio-Rad	1620177
Nonfat dry milk	Apex Bioresearch	20–241

**Experimental models: Cell lines**		

3T3-L1	ATCC	ATCC Cat# CCL-92.1, RRID:CVCL_0123

**Experimental models: Organisms/strains**		

Mouse: C57BL/6, adult, male and female	The Jackson Laboratory	RRID:MGI:2159769

**Other**		

100 μM Sterile cell strainer	Fisherbrand	22-363-549
ThermoMixer F2.0	Eppendorf	EP5387000021

## Materials and equipment

**Table:** NBCS medium (for maintaining of 3T3-L1 cells)

Reagent	Final concentration	Amount
DMEM	N/A	445 mL
NBCS	10%	50 mL
Pen-Strep	1%	5 mL
Total	N/A	500 mL

Store at 4°C for up to 1 month.

**Table:** FBS medium (for differentiation of 3T3-L1 cells)

Reagent	Final concentration	Amount
DMEM	N/A	445 mL
FBS	10%	50 mL
Pen-Strep	1%	5 mL
Total	N/A	500 mL

Store at 4°C for up to 3 month.

**Table:** SVF medium (for maintaining and differentiating SVF)

Reagent	Final concentration	Amount
DMEM/F12	N/A	445 mL
FBS	10%	50 mL
Pen-Strep	1%	5 mL
Total	N/A	500 mL

Store at 4°C for up to 3 month.

**Table:** Mitochondrial extraction buffer (ME buffer)

Reagent	Final concentration	Amount
1 M HEPES (pH 7.8)	20 mM	0.2 mL
1 M NaCl_2_	10 mM	0.1 mL
1.5 M MgCl_2_	1.5 mM	10 μL
0.5 M EDTA	1 mM	20 μL
0.5 M EGTA	1 mM	20 μL
10% Digitonin	0.03%	25 μL
DI water	N/A	9.625 mL
Total	N/A	10 mL

Store at 4°C for up to 3 months. Add protease and phosphatase inhibitors freshly before use.

## Step-by-step method details

### Isolation of crude mitochondria from adipocytes

**Timing: 2 h**

This protocol outlines the isolation of crude mitochondrial and cytosolic fractions from differentiated 3T3-L1 adipocytes and PPDIVs using sequential centrifugation steps (Figure 4). The volume used in this protocol is optimized for a 10 cm dish.

1. Adipocyte harvest.
  a. Wash differentiated adipocytes twice with 5 mL ice-cold PBS.
  b. Scrape the cells from the culture dish into 1 mL of ice-cold ME buffer using a cell scraper.
  c. Transfer the cell suspension to a pre-chilled 1.5 mL microcentrifuge tube.
2. Lyse cells.
  a. Incubate the cell suspension on ice for 20 minutes to allow the cells to swell, which aids in lysis.
  b. Vortex the tube every 5 minutes during the incubation period.
  c. Gently pipette the cell suspension up and down 5–10 times using a P1000 micropipette until the lysate appears milky and no visible cell clumps remain. Avoid excessive shearing forces.

***Note:*** At this stage, you can take a 100 μL aliquot of the lysate and store it at −80°C as a whole cell lysate (WCL) sample. This can be used later to assess the enrichment of mitochondrial and cytosolic fractions by western blotting.

3. Removal of nuclei and debris.
  a. Centrifuge the lysate at 700 × g for 10 minutes at 4°C.
  b. Carefully collect the supernatant, which contains mitochondria and cytosol, and transfer it to a new, pre-chilled 1.5 mL microcentrifuge tube, avoiding the pelleted nuclei and large cellular debris.
  c. Discard the pellet.
  d. Repeat this step twice to prevent nucleus contamination.
4. Isolation of crude mitochondria.
  a. Centrifuge the supernatant from Step 3d at 9,000 × g for 10 minutes at 4°C. The resulting pellet at the bottom of the tube contains the crude mitochondrial fraction.
  b. Carefully transfer the supernatant to a new tube. This supernatant represents the cytosolic fraction.
5. Washing the mitochondrial pellet.
  a. Carefully resuspend the mitochondrial pellet from Step 4a in 1 mL of ice-cold PBS containing 1 mM EDTA and 1 mM EGTA to remove any remaining cytosolic contamination.
  b. Centrifuge the suspension at 9,000 × g for 10 minutes at 4°C.
6. Carefully aspirate and discard the supernatant. The pellet at the bottom of the tube is the washed crude mitochondrial fraction.
7. Processing mitochondrial fraction.
  a. Resuspend the washed mitochondrial pellet in an appropriate volume of ice-cold lysis buffer.
    ***Note:*** For cells harvested from a 10 cm dish, using 100 μL of RIPA lysis buffer typically yields a protein concentration of 1–1.5 μg/μL. Adjust the volume accordingly for different sized dishes or well plates.
  b. Sonicate the resuspended pellet (e.g., 3 pulses of 3 seconds each at moderate power) on ice or pass the lysate through a fine-gauge needle (smaller than 27G) several times to ensure complete lysis of the mitochondria and release of proteins.
  c. Centrifuge the lysate at 16,000 × g for 15 minutes at 4°C to removing any remaining small debris or aggregates. Collect the supernatant as the mitochondrial lysate.
8. Processing cytosolic fraction.
  a. Centrifuge the supernatant collected in Step 4b at 16,000 × g for 15 minutes at 4°C to remove any remaining debris or membrane fragments.
  b. Carefully collect the clarified supernatant and transfer it to a new, pre-chilled microcentrifuge tube. This is your processed cytosolic fraction.
  c. Store the processed mitochondrial lysate and the cytosolic fraction at −80°C for downstream applications.

### Validation of mitochondrial and cytosolic fraction purity by western blotting

**Timing: 2 days**

This step validates the purity and enrichment of mitochondrial and cytosolic fractions obtained from differentiated 3T3-L1 adipocytes or PPDIVs. Immunoblotting for compartment-specific markers ensures reproducibility and confirms successful subcellular fractionation.

9. Sample preparation for western blotting.
  a. Measure protein concentration of WCL, mitochondrial, and cytosolic fractions using a BCA assay.
  b. Normalize samples to equal total protein (e.g., 10–30 μg) in 4x Laemmli Sample Buffer.
  c. Boil samples at 95°C for 5 minutes.
10. SDS-PAGE and transfer.
  a. Load equal amounts of boiled samples protein from WCL, mitochondrial, and cytosolic fractions onto an SDS-PAGE gel.
  b. Run the gel and transfer proteins to a PVDF membrane.
11. Immunoblotting.
  a. Block the membrane in 5% non-fat milk in TBS-T (Tris-buffered saline + 0.1% Tween-20) for 1 hour at 25°C.
  b. Incubate the membrane for at least 12 hours at 4°C with primary antibodies targeting: mitochondrial markers: VDAC1, TOM20, or COXIV, cytosolic markers: β actin or β tubulin, ER marker: Calnexin.
  c. Remove the primary antibodies, wash the membrane 3 times with TBS-T.
  d. Incubate with appropriate HRP-conjugated secondary antibodies diluted in 5% non-fat milk for 1 hour at 25°C with continuous shaking.
  e. Wash 3 times with TBS-T and detect bands using chemiluminescence.

## Expected outcomes

**Figure 3:**
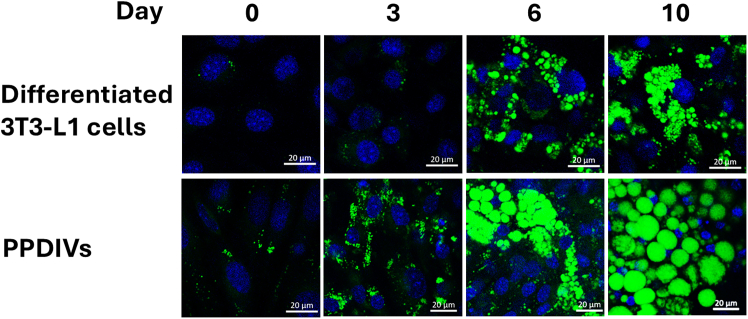
Morphological changes during adipocyte differentiation Representative confocal microscopy images show the progression of adipogenic differentiation over 10 days in differentiated 3T3-L1 cells (top row) and PPDIVs (bottom row). Cells were stained with Hoechst (blue) to label nuclei and BODIPY (green) to label neutral lipid droplets. Images were taken at days 0, 3, 6, and 10 of differentiation. The increase in green fluorescence over time indicates the accumulation of intracellular lipid droplets, characteristic of adipocyte maturation. Scale bars = 20 μm. Images captured at 40× magnification.

This protocol enables the efficient and reproducible differentiation of 3T3-L1 preadipocytes and SVF-derived cells into mature adipocytes (Figure 2), followed by the successful isolation of crude mitochondrial and cytosolic fractions for downstream analysis. During the differentiation process, both 3T3-L1 cells and PPDIVs should exhibit characteristic morphological changes and increase lipid accumulation. Differentiation can be validated by BODIPY staining, which labels neutral lipids. Positive staining is typically observed by Day 6, with increased intensity by Day 10, indicating robust lipid droplet formation (Figure 3).

**Figure 4:**
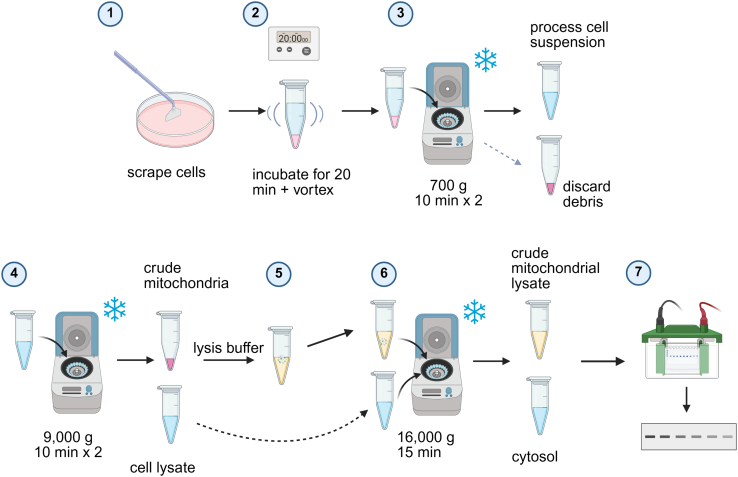
Schematic representation of the crude mitochondrial isolation protocol This figure provides a schematic overview of the sequential centrifugation steps used to isolate crude mitochondrial and cytosolic fractions from differentiated adipocytes. (1) Adherent adipocytes are scraped from the culture dish with 1 mL ME buffer. (2) The resulting cell suspension is incubated on ice for 20 minutes, with intermittent vortexing every 5 minutes, to promote cell swelling and facilitate lysis. (3) The cell lysate is subjected to a low-speed centrifugation at 700 × g for 10 minutes (repeated twice) to pellet nuclei and cellular debris. The supernatant containing mitochondria and cytosol is retained, while the pellet is discarded. (4) The supernatant from step 3 is centrifuged at 9,000 × g for 10 minutes (repeated twice) to pellet crude mitochondria. The resulting supernatant (cell lysate) is saved for cytosolic fraction processing. (5) The crude mitochondrial pellet is resuspended in lysis buffer. (6) Finally, both the mitochondrial lysate and the supernatant from step 4 are centrifuged at 16,000 × g for 15 minutes to clarify the lysates. The resulting supernatants represent the processed mitochondrial lysate and cytosolic fractions, respectively, and are collected for downstream applications. (7) The purity of fractionation is validated by western blot.

The protocol also yields a high number of viable SVF cells from iWAT, suitable for differentiation into adipocytes. When performed correctly, the procedure consistently produces a pure and reproducible SVF population with minimal contamination from mature adipocytes, red blood cells, or tissue debris.

**Figure 5:**
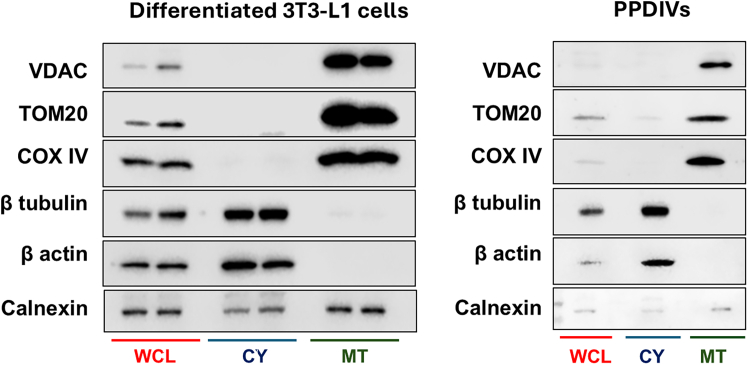
Subcellular fractionation and western blot analysis of adipocytes Western blot analysis was performed on subcellular fractions isolated from differentiated 3T3-L1 cells and PPDIVs to validate the enrichment of specific marker proteins. Fractions analyzed included whole cell lysate (WCL), cytosolic (CY), and crude mitochondrial (MT) preparations. 15 μg of protein from each sample were loaded per lane for western blot analysis. Blots were probed with antibodies against VDAC and TOM20 (mitochondrial outer membrane proteins), COXIV (a mitochondrial inner membrane protein), β-tubulin and β-actin (cytosolic proteins), and calnexin (an endoplasmic reticulum marker). Calnexin detection in MT fractions may indicate mitochondria-associated membranes linked to mitochondrial function and structure. The distribution of these markers across fractions confirms successful enrichment of mitochondrial proteins in the MT fraction and cytosolic proteins in the CY fraction, while also providing insight into the subcellular complexity of crude mitochondrial isolates.

Finally, sequential centrifugation steps result in effective separation of crude mitochondrial and cytosolic fractions. Western blot analysis of these fractions (Figure 5) confirms enrichment of organelle-specific markers, indicating successful subcellular fractionation suitable for downstream biochemical and molecular analyses.

## Limitations

It’s important to acknowledge that this protocol, while useful for adipocyte differentiation and mitochondrial isolation, has certain limitations. Mitochondrial isolation yields a crude fraction, which may have some contamination. For higher purity, alternative methods like density gradient centrifugation are recommended. Furthermore, in vitro models like 3T3-L1 cells and SVF-derived adipocytes may not fully replicate the complex in vivo environment of adipocytes.

## Troubleshooting

### Problem 1

Low yield of SVF after isolation.

### Potential solution

• Incomplete tissue digestion: Thoroughly mince tissue before enzymatic digestion. Optimize collagenase concentration and incubation time based on the equipment used.
• Over digestion: Limit digestion time to ≤30 minutes. Prolonged digestion may reduce cell viability and yield.

### Problem 2

Poor adipocyte differentiation.

### Potential solution

• Suboptimal or degraded reagents: Use freshly prepared differentiation media with validated, endotoxin-free reagents. Mix IBMX well if precipitate forms after freezing. Avoid freeze-thaw cycles for insulin. Test small batches of FBS before large-scale ordering, as variability between lots can affect differentiation efficiency.

### Problem 3

Poor adipocyte differentiation (3T3-L1 cells).

### Potential solution

• Over-passaged cells: Use early-passage cells (ideally <15) and avoid using cells beyond 10 passages post-thaw.
• Overgrown cells used for differentiation: Avoid splitting cells at >80% confluency. Passage cells at 60%–70% confluency to maintain differentiation potential. Monitor growth daily.
• Suboptimal confluency at induction: Allow cells to remain at 100% confluency for 2 days before initiating differentiation.

### Problem 4

Poor adipocyte differentiation (PPDIVs).

### Potential solution

• Over-passaged cells: Use SVF within 2 passages for optimal differentiation. Avoid later passages.
• Insufficient culture time before differentiation: Allow SVF to grow for at least 2 days after reaching confluency before starting differentiation.
• Poor initial SVF health: Do not use SVF that fail to reach confluency within 7 days post-isolation, as they may not differentiate efficiently.

### Problem 5

Contamination of mitochondria in the cytosolic fractions.

### Potential solution

• Excessive lysis time or harsh handling: Limit incubation with lysis buffer to ≤30 minutes. Vortex and gently to prevent mitochondrial rupture and contamination of the cytosolic fraction.

### Problem 6

Contamination of cytosol in the mitochondrial fraction.

### Potential solution

• Insufficient washing of mitochondrial pellet: Wash the mitochondrial pellet at least twice to reduce cytosolic contamination.
• Carryover of unbroken cells from initial centrifugation: Centrifuge the cell suspension at 700 × g at 4°C twice to ensure complete removal of unbroken cells from the supernatant

### Problem 7

Low protein yield from mitochondrial fraction.

### Potential solution

• Insufficient starting material or incomplete lysis: Use at least one confluent 10 cm dish per prep. Incubate cells with lysis buffer for 15–30 minutes to ensure thorough lysis. Check under a microscope if needed.
• Loss of mitochondrial pellet during centrifugation or washing: Avoid disturbing the pellet. Leave a small amount of supernatant when removing or washing to prevent accidental loss.

## Resource availability

### Lead contact

Further information and requests for resources and reagents should be directed to and will be fulfilled by the lead contact, Dr. Alan R. Saltiel (asaltiel@ucsd.edu).

### Technical contact

Technical questions on executing this protocol should be directed to and will be answered by the technical contact, Dr. Churaibhon Wisessaowapak (chwisessaowapak@ucsd.edu).

### Materials availability

This study did not generate new reagents.

### Data and code availability

This study did not generate new datasets or code.

## Acknowledgments

We thank all members of the Saltiel Laboratory for their valuable suggestions. We are also grateful to the staff at the UCSD Microscopy Core for their assistance with the confocal imaging. Special thanks to Dr. Or Benzi and Mr. Mohammad Sedarat from the Scripps Institution of Oceanography for their support with the bright-field microscopy. The UCSD Microscopy core is supported by 10.13039/100000065NINDS P30 NS047101. This work was supported by US NIH (NIH/10.13039/100000062NIDDK) grants P30DK063491, R01DK122804, R01DK124496, R01DK125820, and R01DK128796 to A.R.S.

## Author contributions

Conceptualization, A.R.S. and C.W.; methodology and investigation, C.W., J.L., H.K., S.S., X.F., L.C., A.H., H.C., and S.B.; writing – original draft, C.W.; writing – review and editing, C.W. and S.B.; funding acquisition, A.R.S.; supervision, A.R.S.

## Declaration of interests

The authors declare no competing interests.

## References

[1] Ruiz-Ojeda F.J., Ruperez A.I., Gomez-Llorente C., Gil A., Aguilera C.M. (2016). Cell Models and Their Application for Studying Adipogenic Differentiation in Relation to Obesity: A Review. Int. J. Mol. Sci..

[2] Cai J., Wang F., Shao M. (2023). The Emerging Importance of Mitochondria in White Adipocytes: Neither Last nor Least. Endocrinol. Metab..

[3] Fang C.Y., Wu C.C., Fang C.L., Chen W.Y., Chen C.L. (2017). Long-term growth comparison studies of FBS and FBS alternatives in six head and neck cell lines. PLoS One.

[4] Tang Q.Q., Otto T.C., Lane M.D. (2003). Mitotic clonal expansion: a synchronous process required for adipogenesis. Proc. Natl. Acad. Sci. USA.

[5] Moseti D., Regassa A., Kim W.K. (2016). Molecular Regulation of Adipogenesis and Potential Anti-Adipogenic Bioactive Molecules. Int. J. Mol. Sci..

[6] Tang W., Zeve D., Seo J., Jo A.Y., Graff J.M. (2011). Thiazolidinediones regulate adipose lineage dynamics. Cell Metab..

